# Supplementary material for: Identifying important factors for successful surgery in patients with lateral temporal lobe epilepsy
Source: PLoS One. 2023 Jun 29;18(6):e0288054. doi: 10.1371/journal.pone.0288054 (PMC10310033; doi:10.1371/journal.pone.0288054)
Supplement: Attachment — Submitted filename: Response to Reviewers.docx [file pone.0288054.s003.docx]

Reviewers' comments:

Reviewer's Responses to Questions

**Comments to the Author**

1. Is the manuscript technically sound, and do the data support the conclusions?

The manuscript must describe a technically sound piece of scientific research with data that supports the conclusions. Experiments must have been conducted rigorously, with appropriate controls, replication, and sample sizes. The conclusions must be drawn appropriately based on the data presented.

Reviewer #1: Yes

Reviewer #2: Yes

Reviewer #3: Partly

Reviewer #4: Partly

2. Has the statistical analysis been performed appropriately and rigorously?

Reviewer #1: Yes

Reviewer #2: Yes

Reviewer #3: No

Reviewer #4: I Don't Know

3. Have the authors made all data underlying the findings in their manuscript fully available?

The [PLOS Data policy](http://www.plosone.org/static/policies.action#sharing) requires authors to make all data underlying the findings described in their manuscript fully available without restriction, with rare exception (please refer to the Data Availability Statement in the manuscript PDF file). The data should be provided as part of the manuscript or its supporting information, or deposited to a public repository. For example, in addition to summary statistics, the data points behind means, medians and variance measures should be available. If there are restrictions on publicly sharing data—e.g. participant privacy or use of data from a third party—those must be specified.

Reviewer #1: Yes

Reviewer #2: No

Reviewer #3: Yes

Reviewer #4: Yes

4. Is the manuscript presented in an intelligible fashion and written in standard English?

PLOS ONE does not copyedit accepted manuscripts, so the language in submitted articles must be clear, correct, and unambiguous. Any typographical or grammatical errors should be corrected at revision, so please note any specific errors here.

Reviewer #1: Yes

Reviewer #2: Yes

Reviewer #3: No

Reviewer #4: No

5. Review Comments to the Author

Please use the space provided to explain your answers to the questions above. You may also include additional comments for the author, including concerns about dual publication, research ethics, or publication ethics. (Please upload your review as an attachment if it exceeds 20,000 characters)

**Reviewer #1**: It’s a retrospective clinical study that needs a major revision.
1) Among all 1167 epilepsy surgery patients, 675 patients underwent one-step resection without invasive EEG studies. These 675 patients necessarily included patients with lateral temporal lobe epilepsy. While these 675 patients were excluded from the investigation target, i.e. the 63 lateral temporal lobe epilepsy, which means the conclusion ratio of 71.4% (45/63) seizure free could be inaccurate.
2) The author didn’t mention the exclusion criteria of the study. Since some lateral temporal lobe epilepsy with specific etiologies like tumor and AVM underwent invasive 2-step invasive EEG studies, what kinds of patients should undergo 1-step non-invasive study resection?
3) Since the solitary seizure-free rate of 71.4% (45/63) is not accurate, the author may compare the seizure-free rates between the lateral temporal lobe epilepsy and the mesial temporal lobe epilepsy among all invasive study temporal lobe epilepsy cases (131).

Response for 1-3) Thank you for your comments. We routinely conduct two-step invasive studies except typical patients with mesial temporal lobe epilepsy. Although there are specific lesions in lateral temporal area on MRI, we performed invasive monitoring to find ictal onset zone for tailored resection and minimize risk of surgical failures. We understand your concern, but we are sure include all LTLE patients with inclusion criteria. We excluded patients visited outpatient clinics less than 2 years.

4) The author didn’t clarify the detail of the invasive EEG investigation. Did he implant deep electrodes into the hippocampus in all lateral temporal lobe epilepsy patients? Is it reasonable to do so in epilepsy with a clear lesion?

Response)

Thank you for your comment. We did not implant depth electrodes into hippocampus in all participants. We did it, if other presurgical studies such as functional imaging suggested impairments or alterations in mesial temporal structure. In those cases, we implanted depth to exclude mesio-lateral or mesial TLE and confirmed resection extent. We revised our method section according to your comment.

(page vi, line 134-136)

All participants who were suspected LTLE underwent the invasive monitoring using a combination of subdural grids/strips with or without depth electrodes to confirm the ictal onset zone and determine the extent of resection.

5) The author mentioned that he did anterior temporal lobectomy (ATL) and amygdalohippocampectomy (AH) in some lateral temporal lobe epilepsy patients. So how could he compare LTLE with MTLE since he did the same surgeries for them?
The author should revise the manuscript according to these questions before being considered for publication.

Response)

Thank you for your comment. We did tailored resection of lateral temporal area in all patients and additional ATL and AH in some cases such as combined alterations in imaging. In other words, patients with MTLE underwent standard ATL and AH, but all patients with LTLE underwent tailored corticectomy or lesionectomy. But patients in the subgroup underwent AH simultaneously. So, surgical procedures were not same in LTLE compared to MTLE.

**Reviewer #2**: This is an interesting retrospective cohort study carried out between 1995 and 2018, among patients with lateral temporal lobe epilepsy (LTLE) who underwent resective surgery in a university-affiliated hospital of South Korea. Sixty-four patients were included in the study. The main results were: 1) statistically significant prognostic factors for postsurgical outcomes
were the duration of epilepsy before surgery and focal cortical dysplasia on postoperative histopathology at the 5-year follow-up; 2) cortical thinning was found in ipsilateral cingulate gyrus and contralateral parietal lobe in poor surgical group compared to good surgical group. The Authors also propose a model for predicting seizure outcomes 5 years after surgery using a nomogram.

As a minor observation (at page IX, lines 200-201) the Authors state that "there was no difference between the groups, except for the preoperative disease duration" Anyway, reading Table 1, also follow-up after surgery resulted statistically different in the two groups. So, the Authors should correct this sentence.

There are several typos in the text. For example at page XVIII, line 378, "...better tools are need..." should be changed in " ...better tools are needed..."; at the same page, line 384, "...the ppor surgical..." should be written "...the poor surgical...". The Authors should read agian their manuscript in order to correct typos.

Response) Thank you for your comments. According to your recommendations, we revised some sentences including them.

**Reviewer #3**: This research aimed to evaluate the long-term surgical outcomes and identify possible prognostic factors in patients with lateral temporal lobe epilepsy. Retrospective analysis of sixty-four patients showed FCD was the significant prognostic factors for postsurgical outcomes at the 5-year follow-up. Cortical thinning was observed in ipsilateral cingulate gyrus and contralateral parietal lobe in poor surgical group compared to good surgical group. This study lacked of innovation and interesting. It seems that meaningful conclusions cannot be drawn from the results of this study

I have the following opinions：
1. In the part of abstract, the author should provide a more detailed method such as MRI analysis and clarify conclusions from the results.

Response) Thank you for comments. We revised the abstract.

(page ii, line 30-34)

Methods: This retrospective cohort study was conducted between January 1995 and December 2018 among patients who underwent resective surgery in a university-affiliated hospital. Patients were classified as LTLE if ictal onset zone was in lateral temporal area. Surgical outcomes were evaluated at 2 and 5 years. We subdivided based on outcomes and compared clinical and neuroimaging data including cortical thickness between two groups.

(page ii, line 46-48)

Conclusions: The identified predictors of unfavorable surgical outcomes may help in selecting optimal candidates and identifying the optimal timing for surgery among patients with LTLE. Additionally, cortical thinning was more extensive in the poor surgical group.

2. In the part of cortical thickness, the author may add to MRI-negative image with same age as another control group.

Response) Thank you for comments. We also hoped to analyze cortical thickness compared to healthy controls, but there was no available MRI data. These are our limitations and described in discussion.

(page xx, line 439-440)

Fourthly, the absence of healthy controls in the cortical thickness analysis is another limitation.

3. Based on this study, I think the researchers used voxel based morphology to analyze cortical thickness. If so, voxel-wise FWE correction and threshold with FDR corrected should be used.

Response) Thank you for comments. We used voxel-wise FEW correction and threshold with FDR corrected at first, but we could not get meaningful results. Although the analysis was not ideal, we still believe that we would get significant results in larger participants.

4. A significant English editing is necessary.

Response) Thank you for comments. We already got English editing service, but we checked our manuscript over in general and revised some parts.

**Reviewer #4**: This is a retrospective, single center cohort study aiming to evaluate the short-term and long-term seizure outcomes following resective surgery in patients with lateral temporal lobe epilepsy. This study focused on interesting and important issue in resective epilepsy surgery. However, several limitations unfortunately dampen the enthusiasm for this study.

1. The definition of lateral temporal lobe epilepsy is unclear. It is based on electro-clinical findings? Imaging findings? The authors should clarify.

Response) Thank you for comments. We defined LTLE based on the invasive study. If the seizure onset zone was in lateral temporal area, not mesial temporal structures (hippocampus, amygdala or parahippocampal gyrus) and basal temporal area, we diagnosed as LTLE. We described in method section.

(page iv, line 89-92)

Patients were diagnosed with LTLE if the seizure onset zone was in the lateral temporal area, not in mesial temporal structures or basal temporal area based on an invasive study. The seizure onset zone was defined as any paroxysmal ictal pattern that was distinct from background activity with clinical symptoms.

2. How many parents were underwent invasive EEG diagnostic with subdural or depth electrodes? Were these findings supported by clinical or imaging diagnosis?

Response) Thank you for comments. All enrolled participants were underwent invasive study with subdural electrodes in some cases. We proceeded invasive monitoring, if clinical or imaging diagnosis suggested LTLE. Therefore, presurgical diagnostics supported LTLE and invasive study confirmed it.

3. It is remarkable, that in this study, the long-term seizure outcome is improved compared to the short-term outcome. What is the reason for this observation? The authors should discuss it more extensively.

Response) Thank you for comments. According to your recommendations, we additionally described in our discussion.

(page xvi, line 327-333)

Interestingly, our study showed surgical outcome improved over time. This may be related to study population. As reported by Tellez-Zenteno[24], long-term seizure freedom was highest in patients with tumor, but patients older than 50 years at time of surgery was lowest compared to short-term seizure freedom in temporal lobe surgery. We included patients with tumorous lesions and the average age of surgery was 29.0 years. There are also results that surgical outcomes improved patients with FCD[25], and it is agreement with our results.

4. Since the authors found FCD is significant factor for unfavorable outcome, it is important to know what kind of FCD (classification) was predominantly detected in this cohort.

Response) Thank you for the comment. We tried to report types of FCD, but it was tough. Because more than half of participants were performed epilepsy surgery before 2011, there was no available data of FCD classification. Among available data, 30% (3/10) of each was type 1 and 2 and remaining 40% (4/10) was type 3. So we thought interpretation of it might lead to bias due to limited data, we did not describe it in the manuscript.

5. What is the reason for worse outcome in patients with FCD in this cohort? The authors should explain this noteworthy observation

Response) Thank you for comments. According to your recommendations, we additionally described in our discussion.

(page xvi-xvii, line 347-352)

However, the predictive value of FCD has been controversial, which could be attributed to differences in the study population [8, 27]. Some studies have suggested that complete resection, including dysplasia, is more predictable than FCD itself [28, 29]. This may be one of the reasons why FCD attributes to worse prognosis in our study. In patients with non-lesional MRI, resection margin was determined only by ictal onset zone. It might lead to incomplete resection and seizure recurrence.

6. The subgroup analysis indicates patients with mesial abnormalities and co-existing FCD. This observation is presuming for including of patients with dual pathologies in this study. This could bias both, the findings and the conclusion made by the authors.

Response)

Thank you for comments. Although there were MRI abnormalities in some patients, all ictal onset was seen in lateral temporal areas suggesting LTLE. So, we described reasons of signal changes as seizure related changes or kindling in our discussion.

(page xix, line 408-419)

As confirmed by invasive monitoring, ictal onset zones were in the lateral temporal area in all patients including the subgroup. As a result, signal changes observed in the mesial temporal area could be attributed to other mechanisms. First, seizures can induce signal changes in MRI, which are transient abnormalities that occur after seizure activity in the epileptogenic zone or distinct regions connected by an epileptic network[43]. In some cases, MRI scans were performed after clustering seizures on the last day of EMU monitoring, which could impact the observed signal changes on MRI. Second, the kindling model might be related to these alterations. Kindling refers to a change in seizure characteristics and behaviors resulting from recurrent seizures due to focal electrical stimulation.[44] Limbic circuits, including the hippocampus and amygdala, are highly vulnerable to kindling, which can eventually lead to epileptogenicity in affected regions. One study showed changes observed in the mesial temporal area may be due to the kindling process.[45]

6. PLOS authors have the option to publish the peer review history of their article ([what does this mean?](https://journals.plos.org/plosone/s/editorial-and-peer-review-process#loc-peer-review-history)). If published, this will include your full peer review and any attached files.

If you choose “no”, your identity will remain anonymous but your review may still be made public.

**Do you want your identity to be public for this peer review?** For information about this choice, including consent withdrawal, please see our [Privacy Policy](https://www.plos.org/privacy-policy).

Reviewer #1: No

Reviewer #2: No

Reviewer #3: No

Reviewer #4: No

[NOTE: If reviewer comments were submitted as an attachment file, they will be attached to this email and accessible via the submission site. Please log into your account, locate the manuscript record, and check for the action link "View Attachments". If this link does not appear, there are no attachment files.]

While revising your submission, please upload your figure files to the Preflight Analysis and Conversion Engine (PACE) digital diagnostic tool, <https://pacev2.apexcovantage.com/>. PACE helps ensure that figures meet PLOS requirements. To use PACE, you must first register as a user. Registration is free. Then, login and navigate to the UPLOAD tab, where you will find detailed instructions on how to use the tool. If you encounter any issues or have any questions when using PACE, please email PLOS at [figures@plos.org](mailto:figures@plos.org). Please note that Supporting Information files do not need this step.
